# Indirect Comparison of 10 kHz Spinal Cord Stimulation (SCS) versus Traditional Low-Frequency SCS for the Treatment of Painful Diabetic Neuropathy: A Systematic Review of Randomized Controlled Trials

**DOI:** 10.3390/biomedicines10102630

**Published:** 2022-10-19

**Authors:** Bryan C. Hoelzer, Deborah Edgar, Shiao-Ping Lu, Rod S. Taylor

**Affiliations:** 1 Medical Director, Southwest Spine and Pain Center, Provo, UT 84059, USA; 2 Commexus Ltd., Dunblane FK15 0DF, UK; 3 Lucid Consulting, Los Altos, CA 94024, USA; 4 MRC/CSO Social and Public Health Sciences Unit & Robertson Centre for Biostatistics, School of Health and Well Being, College of Medical, Veterinary and Life Sciences, University of Glasgow, Glasgow G12 8QQ, UK; 5 Health Service Research, College of Medicine and Health, University of Exeter, Exeter EX1 2LU, UK; 6 National Institute of Public Health, University of South Denmark, 1455 Copenhagen, Denmark

**Keywords:** painful diabetic neuropathy, peripheral neuropathy, spinal cord stimulation, 10 kHz SCS, diabetes, neuropathic pain, systematic review, meta-analysis

## Abstract

Spinal cord stimulation (SCS) is increasingly used to treat painful diabetic neuropathy (PDN). At the time of a recent meta-analysis in this field, data were only available from randomized controlled trials (RCTs) of traditional low-frequency SCS (LF-SCS). However, outcomes from high-frequency 10 kHz SCS treatment are now available. Our study aimed to systematically review the contemporary evidence for SCS in patients with lower limb pain due to PDN and include an indirect comparison of the high- and low-frequency modalities. We searched the PubMed/CENTRAL databases up to 18 August 2022, for peer-reviewed RCTs of SCS that enrolled PDN patients with lower limb pain symptoms. The quality of the evidence was assessed with the Cochrane Risk of Bias tool. Using SCS treatment arm data from the RCTs, we indirectly compared the absolute treatment effect of 10 kHz SCS and LF-SCS. Results are presented in tables and forest plots. This systematic review was reported according to the Preferred Reporting Items for Systematic reviews and Meta-Analyses (PRISMA) 2020 guidelines. Three RCTs met our eligibility criteria, including the recent 10 kHz SCS RCT (*N* = 216, 90 implanted) and 2 others that examined LF-SCS (*N* = 36, 17 implanted; *N* = 60, 37 implanted). Our analysis of 6-month data found clinically meaningful pain relief with each SCS modality. However, significantly greater pain reduction was identified for 10 kHz SCS over LF-SCS: average pain reduction in the 10 kHz SCS cohort was 73.7% compared with 47.5% in the pooled LF-SCS group (*p* < 0.0001). In the permanent implant subset, the 50% pain reduction responder rate was 83.3% in the 10 kHz SCS cohort versus 63.0% in the pooled LF-SCS group (*p* = 0.0072). The overall risk of bias of each included RCT was deemed high, mainly due to the absence of patient blinding. Our analysis indicates that paresthesia-free 10 kHz SCS can provide superior pain relief and responder rate over LF-SCS for managing PDN patients refractory to conventional medical management.

## 1. Introduction

Diabetic neuropathy (DN) is the most common complication of diabetes, affecting approximately half of all patients [1,2]. The condition is characterized by a chronic sensory loss that gradually progresses proximally from the feet and hands in a glove-and-stocking pattern [1]. In addition, approximately 30% of DN patients develop neuropathic pain, a condition referred to as painful diabetic neuropathy (PDN). Typically, PDN presents as burning pain with concurrent paresthesia, resulting in a poor health-related quality of life (HRQoL), depression, anxiety, and impaired sleep [1,3,4,5].

Pharmacotherapy is the mainstay of analgesic treatment for PDN, with clinical guidelines recommending pregabalin and duloxetine as first-line treatment options [6]. Other recommended agents include gabapentin and tricyclic antidepressants (principally amitriptyline) [6]. While these drugs may help manage neuropathic pain, their efficacy is limited [7]. In addition, side effects are often significant, leading to poor adherence or discontinuation, even among those with initial treatment success [8,9].

Since the late 1960s, traditional low-frequency spinal cord stimulation (LF-SCS) has been a recognized treatment for chronic neuropathic pain. During LF-SCS, therapeutic paresthesia is elicited in the painful dermatomes by applying electrical pulses to the spinal cord at frequencies ranging from 40 to 60 Hz. Successful masking of pain relies on concordance of the induced paresthesia with the painful area. The therapy is a common treatment option for postlaminectomy syndrome and complex regional pain syndrome [10]. However, the continuous perception of paresthesia during LF-SCS can be uncomfortable [11,12], and only around half of treated patients experience adequate pain relief [11,13,14].

Over the last decade, novel SCS waveforms have emerged that provide pain relief at subsensory levels, which may be a more comfortable and desirable option for patients. One such stimulation paradigm is high-frequency SCS at 10 kHz (10 kHz SCS), a paresthesia-free therapy whose mechanism of action is independent of paresthesia [15]. Compared with LF-SCS, the high-frequency modality provided superior relief of back and leg pain in a randomized controlled trial (RCT), with a significantly higher proportion of 10 kHz SCS individuals with chronic back pain reporting response to therapy at long-term follow-up (76.5% vs. 49%, *p* < 0.001) [11,16]. In addition, promising outcomes with the high-frequency modality have been observed in other neuropathic pain syndromes [17,18,19,20,21,22,23,24,25].

Several pilot studies have also explored SCS as a treatment for PDN refractory to conventional medical management (CMM), with encouraging outcomes across all modalities [26,27,28,29,30,31,32,33,34,35,36,37]. Subsequent RCTs provided high-quality evidence supporting the use of LF-SCS and 10 kHz SCS in this patient group [38,39,40]. However, long-term follow-up data (5 years) suggest that the effectiveness of LF-SCS diminishes over time [39,41,42], a phenomenon also documented in other neuropathic pain conditions [12,27,43,44,45,46,47,48,49]. More recently, analyses of 6- and 12-month follow-up data from the SENZA-PDN trial showed that 10 kHz SCS provided sustained pain relief over time compared with CMM [50]. Studies in other chronic pain syndromes also suggest that the therapy is effective and durable after several years [11,16,51,52,53,54].

A recent systematic review and meta-analysis assessed the effectiveness of SCS for the management of PDN [55]. At the time of the analysis, data were only available from RCTs that compared LF-SCS with CMM. Therefore, our study aimed to systematically review the contemporary RCT evidence for SCS in patients with lower limb pain due to PDN and include an indirect comparison of 10 kHz SCS and LF-SCS treatment outcomes. Our analysis may be valuable to clinicians and patients during their evaluation of different SCS modalities.

## 2. Materials and Methods

### 2.1. Eligibility

Articles were eligible for inclusion if they reported pain-related outcomes from an RCT of SCS that enrolled PDN patients with lower limb pain symptoms. Publications were excluded if (1) they were not peer-reviewed or had no full-text manuscript available (e.g., conference proceedings), (2) no original data were presented (e.g., data duplication, protocol and technical descriptions, commentaries, and review articles), or (3) data could not be extracted for the population of interest (e.g., PDN subgroup data were not separated).

### 2.2. Search Strategy

We searched the PubMed and CENTRAL electronic databases from inception to 18 August 2022, using a combination of MESH terms and free-text words. Additional published filters were used to identify RCTs during the PubMed search [56,57]. The detailed search strategy is presented in Supplementary Material 1 (Tables S1 and S2).

### 2.3. Selection Process

A single reviewer (D.E.) screened titles and abstracts to identify articles eligible for further review. Full-text manuscripts were subsequently obtained and assessed for compliance with eligibility criteria.

### 2.4. Data Extraction and Outcomes

Two reviewers (S.L. and D.E.) extracted the following data from the eligible RCT publications: study design variables, patient demographics, sample size, interventions, mean pain intensity scores measured using either the visual analog scale (VAS; 0–10 cm or 0–100 points) or a numerical rating scale (NRS; 0–10 points), number of subjects with at least a 50% reduction in pain intensity from baseline (i.e., a responder), and treatment-related adverse events (AEs). In addition, SL extracted patient-level pain intensity scores if the individual patient data (IPD) were available.

Two standard SCS efficacy outcomes were used in our analysis: (1) mean pain intensity reduction from baseline and (2) responder rate, i.e., defined as the proportion of subjects with at least a 50% reduction in pain intensity from baseline.

### 2.5. Study Risk of Bias Assessment

A single reviewer (D.E.) assessed the risk of bias for each eligible RCT using the Cochrane Risk of Bias 2 (RoB 2) tool, according to the criteria outlined in the *Cochrane Handbook for Systematic Reviews of Interventions* [58]. The RoB 2 tool included assessment across multiple domains: (1) bias arising from the randomization process; (2) bias due to deviations from intended interventions; (3) bias due to missing outcome data; (4) bias in measurement of the outcome; and (5) bias in selection of the reported result. Each domain was graded as low risk, high risk, or with some concerns.

### 2.6. Measures of Treatment Effect

We used the mean difference (MD) with a 95% confidence interval (CI) for the continuous outcome of pain intensity reduction from baseline. Relative risk (RR) with a 95% CI was chosen for the dichotomous responder rate outcome.

### 2.7. Statistical Analysis

We conducted an indirect comparison of the absolute treatment effect of 10 kHz SCS and LF-SCS using SCS treatment arm data from the included RCTs (10 kHz SCS: Petersen et al. [38]; LF-SCS: Slangen et al. [39] and de Vos et al. [40]).

The analysis was performed at 6 months postimplantation because this was the longest follow-up duration common to all 3 RCTs. We did not anticipate clinical heterogeneity as all interventions were a type of SCS applied in a similar population under comparable clinical trial conditions. Statistical analyses were performed in SAS, with individual study outcomes and study comparisons presented in tables and forest plots.

#### 2.7.1. Analysis Populations

We first created 4 groups from the 3 included RCTs: (1) 10 kHz SCS [38], (2) Slangen LF-SCS [39], de Vos LF-SCS [40], and (4) a pooled LF-SCS group that combined outcomes from the de Vos and Slangen studies.

Two analysis populations were defined for each group that allowed the comparison of equivalent patient cohorts: (1) a modified intention-to-treat (mITT) population consisting of randomized individuals who entered the SCS trial phase and (2) a permanent implant population with subjects who completed the SCS trial phase and received a permanent system.

#### 2.7.2. Mean Pain Intensity Reduction

Next, we determined the mITT mean pain intensity reductions with 95% CI at 6 months for each of the 4 groups, including data conversion as needed to allow consistent comparison between studies (as per the Duarte et al., meta-analysis [55]). The mean pain intensity reduction for the 10 kHz SCS group was derived from the mITT individual patient data (IPD), which allowed the calculation of individual absolute pain intensity reduction at 6 months. This analysis imputed missing values using the last observation carried forward (LOCF), akin to the method employed in the Slangen and de Vos studies.

Data for the Slangen LF-SCS group were sourced directly from the study publication. The investigators assessed day- and night-time pain separately and presented outcomes at the aggregated subject level. From these data, we calculated an average (with pooled variance) of the 0–10 NRS daytime and night-time pain scores (pain scores and 95% CI were derived from Figure 2 in the Slangen publication using WebPlotDigitizer, https://automeris.io/WebPlotDigitizer, accessed 25 August 2022). For the de Vos LF-SCS group, we extracted mean pain scores directly from the study publication. Since the investigators assessed pain using a 0-100 VAS, we divided the mean pain scores by 10. Data from the Slangen and de Vos studies were combined for the pooled LF-SCS group, with the pooled mean pain reduction and variance determined via the inverse variance method.

We subsequently compared the 10 kHz SCS pain reduction with each LF-SCS group using the two-sample *t*-test. In addition, we performed a test for the assumption of equal variance, with the Satterthwaite method used to derive the pooled variance if we found the variance to be unequal.

#### 2.7.3. Responder Rate

We also determined responder rates and RRs with 95% CI for the mITT and permanent implant populations at 6 months for each of the 4 groups, i.e., 10 kHz SCS, Slangen LF-SCS, de Vos LF-SCS, and pooled LF-SCS. The respective responder rates for the 10 kHz SCS group were calculated from the study IPD. For the Slangen and de Vos studies, we extracted the responder data directly from the study publications. Individuals who discontinued treatment early due to cause were classified as SCS failures (i.e., nonresponders). We averaged the day- and night-time responder rates from the Slangen study. For the pooled LF-SCS group, we combined the responder data from the Slangen and de Vos study publications. The pooled relative risk for the responder rates and associated confidence intervals were calculated using the Mantel-Haenszel method.

We subsequently compared the population-level responder rates between the 10 kHz SCS cohort and each LF-SCS group. These comparisons used the Wald Chi-square statistic under the null hypothesis of RR = 1, i.e., no difference between groups.

#### 2.7.4. Adverse Events

Given the varied presentation methods used in the included studies, formal numeric synthesis of study-related AEs was not applicable. Instead, we tabulated and narratively summarized the AE findings across the included trials.

## 3. Results

### 3.1. Study Selection

**Figure 1:**
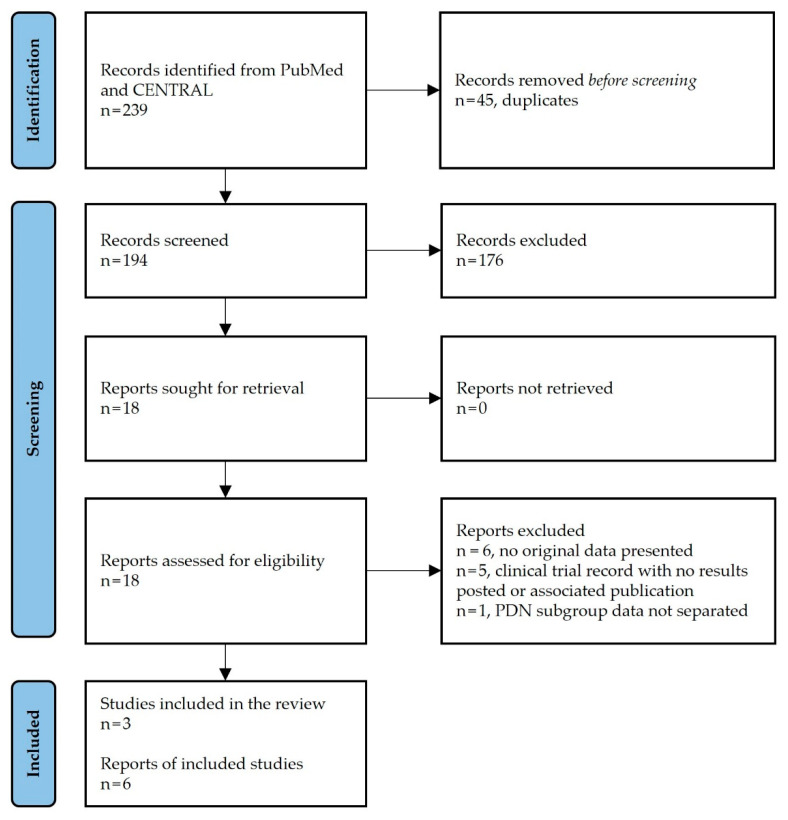
Flow chart of the systematic literature search according to PRISMA guidelines. Registration number: INPLASY202290056. DOI: 10.37766/inplasy2022.9.0056.

The search strategy retrieved 194 unique citations (Figure 1). Of these, 176 were excluded based on title and abstract content. After a full-text assessment of the remaining 18 citations, 6 articles met the eligibility criteria. These included 3 individual RCT reports by Petersen et al. [38], Slangen et al. [39], and de Vos et al. [40], and 3 additional publications of extended follow-up data from the Petersen and Slangen RCTs [41,42,50].

### 3.2. Study Characteristics

Study characteristics are summarized in Table 1. The included RCTs were conducted in multiple centers, either in the USA [38], the Netherlands [39], or internationally (the Netherlands, Denmark, Belgium, and Germany) [40]. Stimulation-treated subjects in the Petersen study received 10 kHz SCS, a modality that provides pain relief without paresthesia (Nevro Corp. [38]). In the remaining 2 RCTs, participants assigned to LF-SCS received a study-specific manufacturer device that induced continuous paresthesia to mask the sensation of pain (Medtronic [39] and St. Jude Medical [40]).

Among the studies, all subjects had PDN with predominant lower limb or lower extremity pain (VAS or NRS ≥ 5 on a 0–10 scale) refractory to previous treatments; however, the definition of refractory varied. In addition, significant upper limb or upper extremity pain symptoms were an exclusion criterion in all studies.

All 3 RCTs had an open-label, parallel-group design with a total of 312 participants (range 36 to 216) randomized in differing ratios (1:1 [38], 3:2 [39], and 2:1 [40]). The largest study by Petersen and colleagues compared CMM with or without adjunctive 10 kHz SCS [38]. In the remaining 2 studies, Slangen et al., and de Vos et al., compared best medical treatment (BMT, which is consistent with and now referred to as CMM) with or without adjunctive LF-SCS [39,40]. Common to all studies was a screening trial for subjects assigned to SCS and a 6-month duration parallel-group phase. In addition, participants had the option to cross over to the other arm after the 6-month randomized phase in all 3 RCTs; however, only 2 studies documented crossover criteria in the initial publication [38,40]. Of the 3 RCTs, 2 had extended follow-up data [41,42,50].

Across the 3 studies, the mean age of participants was similar, as was the distribution of sex between groups, the predominance of type 2 diabetes, and the long-term duration of both diabetes and painful symptoms/peripheral neuropathy. However, 1 study had proportionately more type 1 diabetes participants than the other studies [40], and another had a shorter PDN duration [39].

All studies reported adverse events (AEs). However, the level of detail varied. For example, the Slangen RCT presented only serious adverse events (SAEs) during their 6-month randomized phase [39]. In contrast, Petersen and colleagues documented all study-related AEs during the same period [38]. In addition, the extended follow-up reports [41,42] for the Slangen study provided more comprehensive AE details than the earlier publication [39], with the final analysis of 12- to 60-month data including 33 of the 36 randomized patients and an additional 15 subjects from another pilot study by Pluijms et al. (total: *n* = 48 trialed; *n* = 40 permanent implants) [31].

### 3.3. Risk of Bias in Studies

**Figure 2:**
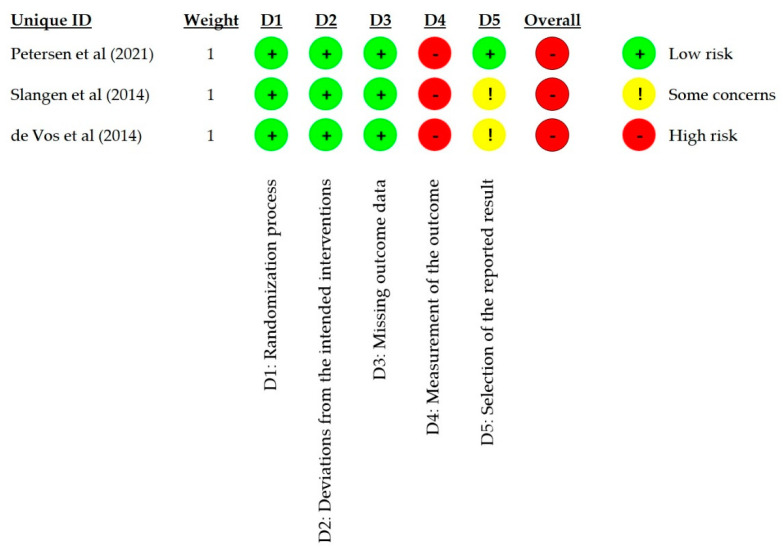
Risk of bias assessment using the Cochrane RoB—2 tool. Study ID: Petersen et al. (2022) [38], Slangen et al. (2014) [39], de Vos et al. (2014) [40].

Figure 2 summarizes the risk of bias assessment for the included RCTs, with the full details available in Supplementary Material 2, Table S3.

We judged all RCTs to have a low risk of bias for the randomization process (D1) and deviations from the intended interventions (D2).

In the missing outcome data assessment (D3), we judged all 3 RCTs to have a low risk of bias at 6 months for different reasons. For example, despite an imbalance in the levels of missing data between the groups in the Slangen RCT, the levels of missing data were low, and the analysis designated all missing subjects as nonresponders [39]. At the same time, the de Vos RCT had a low and balanced level of missing data and an ITT analysis approach [40]. In the Petersen RCT, the level of missing data in each arm was also low but imbalanced between the groups [38]. The 6 individuals who failed the screening trial (i.e., pain reduction < 50%) were considered nonresponders; however, 8 additional subjects with a successful trial (i.e., pain reduction ≥ 50%) dropped out for other reasons. Since the 6-month analysis omitted the latter individuals, we examined their IPD to assess whether the missingness of the data was dependent on its true value. We concluded that the missingness of the data was probably not pain-related, leading to a low risk of bias judgment for D3.

We deemed all 3 studies to have a high risk of bias in the fourth domain (D4) due to the use of a patient-reported outcome (PRO) in an unblinded study, i.e., the assessor was aware of the treatment allocation. Open-label study designs, such as those used in all 3 RCTs, are standard in the SCS field due to the need for an implanted device and perceptible paresthesia during LF-SCS. Using validated pain PROs, such as VAS and NRS, is also typical. Considering the design of the included RCTs, the pain PRO could have been influenced by an expected benefit from permanent stimulation or CMM not perceived as proper treatment. Furthermore, participants in all 3 studies knew they could cross to the other treatment arm after 6 months if their treatment was inadequate.

While there were no apparent indications of selective data reporting in the Slangen or de Vos studies, neither had a published protocol or statistical analysis plan (SAP) [39,40], leading to some concern of potential bias in the fifth domain (D5). In contrast, a protocol summary and SAP were available for the Petersen RCT [59,60]. After examining both documents, we judged the study to be at low risk of selective data reporting.

The overall risk of bias was deemed high for each RCT due to the high risk of bias rating in D4, primarily due to the studies using a PRO in the absence of patient blinding.

### 3.4. Pain Intensity Outcomes

**Figure 3:**
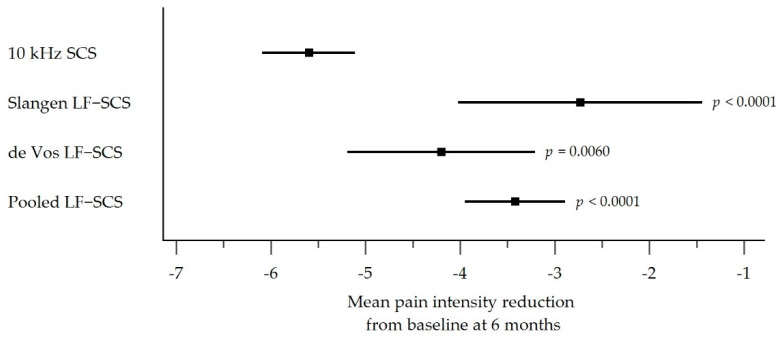
Forest plot comparison of the mITT population mean pain intensity reductions from baseline at 6 months with 95% CI (*p*-value is for 10 kHz SCS vs. LF-SCS group). The mITT population comprised randomized individuals who entered the SCS trial phase. Abbreviations: CI, Confidence Interval; LF-SCS, Low-Frequency Spinal Cord Stimulation; mITT, Modified Intention-to-Treat; SCS, Spinal Cord Stimulation.

Table 2 shows the mITT pain intensity reduction from baseline for the 10 kHz SCS cohort and each LF-SCS group (Slangen LF-SCS, de Vos LF-SCS, and pooled LF-SCS). Individuals treated with 10 kHz SCS had the largest reduction in pain from baseline at 6 months (−5.60 points, 95% CI −6.09 to −5.11; 73.7% reduction) compared with the Slangen LF-SCS group (−2.73 points, 95% CI −4.02 to −1.44; 38.7% reduction), de Vos LF-SCS group (−4.20 points, 95% CI −5.19 to −3.21; 57.5% reduction), and the pooled LF-SCS group (−3.42 points, 95% CI (−3.95 to −2.89; 47.5% reduction). All 3 comparisons were highly statistically significant, indicating that 10 kHz SCS provided superior pain reduction versus LF-SCS (*p* < 0.0001 vs. Slangen LF-SCS; *p* = 0.0060 vs. de Vos LF-SCS; *p* < 0.0001 vs. pooled LF-SCS; Table 2 and Figure 3).

### 3.5. Responder Rate Outcomes

**Figure 4:**
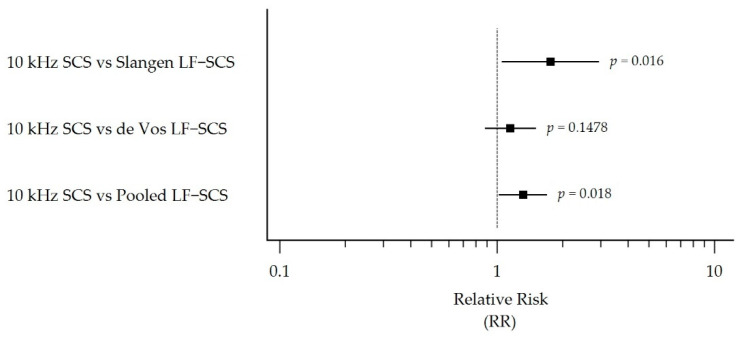
Forest plot comparison of the mITT population responder rates at 6 months, showing RR with 95% CI. The mITT comprised randomized individuals who entered the SCS trial phase. Abbreviations: CI, Confidence Interval; LF-SCS, Traditional Low-Frequency Spinal Cord Stimulation; mITT, Modified Intention-to-Treat; RR, Relative Ratio; SCS, Spinal Cord Stimulation.

Table 3 presents the mITT responder rate outcomes for the 10 kHz SCS cohort and each LF-SCS group (Slangen LF-SCS, de Vos LF-SCS, and pooled LF-SCS). Subjects treated with 10 kHz SCS were 1.76 (95% CI 1.05 to 2.95) and 1.32 (95% CI 1.02 to 1.70) times more likely to respond to therapy than the Slangen LF-SCS group (*p* = 0.016) and the pooled LF-SCS group (*p* = 0.018), respectively (Table 3 and Figure 4). While favoring 10 kHz SCS, the difference between the 10 kHz SCS and de Vos LF-SCS groups did not reach statistical significance (RR 1.15, 95% CI 0.88 to 1.51, *p* = 0.1478).

**Figure 5:**
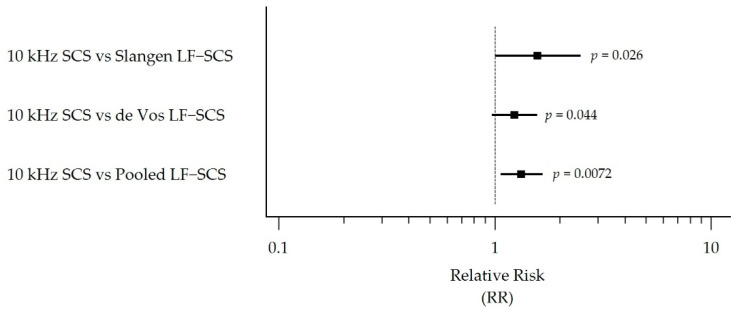
Forest plot comparison of the permanent implant population responder rates at 6 months, showing RR with 95% CI. The permanent implant population comprised subjects who completed the SCS trial phase and received a permanent system. Abbreviations: CI, Confidence Interval; LF-SCS, Traditional Low-Frequency Spinal Cord Stimulation; RR, Relative Risk; SCS, Spinal Cord Stimulation.

The corresponding outcomes and RRs for the permanent implant populations are shown in Table 4 and Figure 5. In these populations, subjects treated with 10 kHz SCS were 1.57 (95% CI 1.00 to 2.49), 1.23 (95% CI 0.97 to 1.57), and 1.32 (95% CI 1.06 to 1.66) times more likely to respond to therapy than the Slangen LF-SCS group (*p* = 0.026), de Vos LF-SCS group (*p* = 0.044), and pooled LF-SCS group (*p* = 0.0072), respectively.

### 3.6. Treatment-Related Adverse Events

Table 5 summarizes treatment-related AEs extracted from the included RCTs and subsequent follow-up reports. In the Petersen RCT, 18 treatment-related AEs occurred in 14 patients during the first 6 months of follow-up. Among these were instances of postimplant infection (*n* = 3), wound dehiscence (*n* = 2), impaired wound healing (*n* = 1), device extrusion (*n* = 1), incision site pain (*n* = 1), implantable pulse generator (IPG) site pain (*n* = 1), lead migration (*n* = 1), and uncomfortable stimulation (*n* = 1) [38]. The investigators classified 2 of the AEs as serious, with 2 subjects requiring explant. After 12 months of treatment, the investigators documented 8 postimplant infections and 5 subsequent explants among participants initially treated with 10 kHz SCS and those who received the therapy after crossover at 6 months [50]. In addition, there were 2 instances of IPG revision and 1 of lead migration with subsequent revision. No IPG replacements were reported during the 6- or 12-month follow-up periods.

Slangen and colleagues documented 2 SAEs during their 6-month randomization phase, including 1 death from a subdural hematoma following a dural puncture and 1 postimplant infection leading to explant [39]. No IPG replacements were noted during this study phase. By the last published follow-up of the Slangen study at 60 months, which included 15 additional patients [31], 10 subjects had reported IPG site pain, 9 had experienced uncomfortable stimulation, and 2 had developed postimplant infections that led to device removal. In addition, 4 individuals underwent lead revision to optimize paresthesia coverage, 3 had damaged leads replaced, 1 had their IPG relocated due to prolonged pocket pain, 6 had their system explanted due to lack of efficacy, 8 required a single battery replacement, and 5 required 2 battery replacements [42].

The investigators in the de Vos study reported several treatment-related AEs during their 6-month RCT. These included the repositioning of 1 lead due to migration and 2 leads due to poor paresthesia overlap, the revision of 2 IPGs due to pain around the implant site, 1 infection during the trial phase, and 1 coagulopathy during the permanent implantation procedure [40].

## 4. Discussion

### 4.1. Interpretation of Results

Our literature search identified 3 RCTs that assessed the impact of SCS in patients with lower limb pain due to PDN. One study reported outcomes from 10 kHz SCS treatment, a modality that provides pain relief without paresthesia (SENZA-PDN study by Petersen et al. [38]). The remaining 2 studies presented LF-SCS results, with each study using a specific manufacturer’s device that induced continuous paresthesia (Slangen et al. [39] and de Vos et al. [40]).

Overall, our analysis found a mean reduction in pain intensity from baseline that exceeded the minimal clinically important difference (MCID) of 2 points or a 30% reduction, regardless of SCS modality [61]. However, our mITT comparison of mean pain score reductions between modalities showed a significant effect in favor of 10 kHz SCS over LF-SCS: Individuals treated with 10 kHz SCS had a 5.6-point reduction in mean pain score from baseline (2.8 times the MCID) compared with a 3.4-point decrease in the pooled LF-SCS group (1.7 times the MCID; *p* < 0.0001).

In all but one scenario of analysis population, individuals treated with 10 kHz SCS were also significantly more likely to respond to therapy at 6 months postimplantation than subjects who received LF-SCS.

In summary, patients with refractory PDN who received 10 kHz SCS had a significantly higher reduction in pain and a greater likelihood of response to therapy at 6 months than those who received LF-SCS. In addition, 12-month results from the SENZA-PDN study showed sustained pain relief and replication of outcomes in crossover patients [50], with 24-month data expected after study completion.

Our findings support previous research that showed the superiority of 10 kHz SCS over LF-SCS in chronic back and leg pain [11,16]. In the SENZA-RCT, recipients of 10 kHz SCS were 1.5 times (95% CI 1.2 to 1.9) more likely to respond to therapy at 6 months than those treated with LF-SCS, with the RR sustained at 12 months. After 24 months, the reduction in mean pain intensity from baseline remained significantly larger in the 10 kHz SCS group compared with the LF-SCS cohort (back pain MD −1.7, 95% CI −2.6 to −0.8, *p* < 0.001). While the patient populations in the SENZA-RCT and the SENZA-PDN studies differed, the higher level of pain relief provided by 10 kHz SCS in both patient groups suggests that the high-frequency modality may be a more robust pain therapy than LF-SCS.

In general, SCS is a well-recognized treatment option for patients with neuropathic pain symptoms, with the therapy increasingly used in the PDN indication. Our results suggest that 10 kHz SCS may improve clinical outcomes over LF-SCS in this patient group, an important finding in a difficult-to-treat cohort. Furthermore, the need for effective nonpharmacological treatments for this indication will only increase, given the rising prevalence of diabetes worldwide [62].

The difference in outcomes between the high- and low-frequency stimulation modalities may be due to differing mechanisms of action (MOA). The analgesic effect of LF-SCS is premised on the gate control theory of pain, which postulates that the transmission of pain signals can be modulated by inhibitory interneurons in the spinal dorsal horn activated by antidromic stimulation of Aβ fibers in the dorsal column [63,64,65]. These interneurons also mediate pain by enhancing release of the inhibitory neurotransmitter GABA (gamma-aminobutyric acid). LF-SCS may also modulate the processing of pain signals through the spinothalamic tract [64,65]. Meanwhile, simultaneous orthodromic stimulation of the dorsal column Aβ fibers during LF-SCS generates paresthesia in the area innervated by the fibers [64,65].

The absence of paresthesia during 10 kHz SCS suggests that its pain-relieving effect is not dependent on the activation of dorsal column Aβ fibers. This theory is supported by preclinical research that suggests low-intensity 10 kHz stimulation activates inhibitory interneurons in the dorsal horn without activating the dorsal column fibers that cause paresthesia [66]. High-frequency stimulation has also been observed to partially normalize spinal glutamate uptake activity, spinal glutamate levels, and miniature excitatory postsynaptic current frequency in rats with spared nerve injury-induced neuropathic pain [67]. A recent investigation of low-intensity 10 kHz SCS in diabetic rodent models demonstrated not only behavioral measures of reduced mechanical hypersensitivity (analog of clinical pain relief), but also a normalization of the receptive fields of dorsal horn neurons, thus reducing central sensitization [68]. This result suggests that 10 kHz SCS may work through predominantly inhibitory spinal mechanisms, in contrast to low-frequency paresthesia-based SCS, which non-selectively drives both excitatory and inhibitory spinal interneurons [69].

In support of a different MOA, in a head-to-head clinical trial (SENZA-RCT), 10 kHz SCS provided superior relief of back and leg pain over LF-SCS, with a significantly higher proportion of 10 kHz SCS individuals with chronic back pain reporting response to therapy at long-term follow-up (76.5% vs. 49.3%, *p* < 0.001) [11,16]. Accordingly, clinical data show superior pain relief in patients with back pain as well as in PDN patients, as demonstrated in this review. Both the SENZA-RCT and our results support that the MOA of 10 kHz SCS is unique and more robust for the treatment of chronic pain conditions.

The absence of paresthesia during 10 kHz SCS may improve patient comfort and therapy acceptability in general, and may be especially beneficial in PDN patients who often have underlying disease-related paresthesia symptoms. Moreover, 10 kHz SCS may improve neurological deficits associated with PDN, an outcome that does not appear to have been assessed in the LF-SCS studies. Petersen et al., reported that 62% of patients experienced a clinician-assessed neurological improvement after 6 months of 10 kHz SCS treatment, with most improvements observed in sensory function [38]. Taken together, 10 kHz SCS may provide additional patient benefits beyond pain relief.

### 4.2. Treatment-Related Adverse Events

Complications arising from lead displacement, lead breakage, and pain around the implant site are well described during SCS, with respective incidence rate ranges of 2% to 27%, 6% to 10%, 1% to 12% (excluding PDN studies) [70]. However, most complications are resolved despite often requiring revision surgery [71].

Although all studies included in our review reported AE data, the number of studies was small, and the level of detail varied. Overall, the reported complication rates associated with leads and IPGs were broadly comparable to the SCS literature. Importantly, this was also true for the incidence of infection, which is of particular interest in PDN patients, given the susceptibility of diabetic patients to infection [72]. The general SCS literature (excluding PDN studies) estimates that the incidence of infection ranges from 3% to 10% [70]. In our review, we found short-term (6-month) postimplantation infection rates of 3.3% in the Petersen study (3 of 90), 5.9% in the Slangen RCT (1 of 17), and nil in the de Vos study (0 of 37, although the de Vos investigators noted 1 infection during the trial phase). Longer-term, the equivalent incidence rates in the Petersen and Slangen studies were both approximately 5% (8 of 154 and 2 of 40, respectively). The comparability of SCS infection rates in PDN populations to other patient groups is a notable finding amid the continuing debate about whether diabetes is a risk factor for infection during SCS [73,74,75]. However, more extensive studies are required to establish whether PDN patients are more at risk of infection than other SCS candidates.

### 4.3. Strengths and Limitations of the Review

This review is the first to compare 10 kHz SCS with LF-SCS for the treatment of PDN. While we did not prepare a predefined protocol or register the systematic review, we conducted structured searches and analyses. In addition, we provided a detailed and transparent presentation of methods and data to allow reproducibility. We also reported the study in line with the PRISMA 2020 guidelines for systematic reviews [76].

The small number of RCTs included in our review should be borne in mind when interpreting our results. In addition, the control arm in each included study was CMM, which prevented a direct comparison of 10 kHz SCS and LF-SCS. However, as the individual RCTs were broadly similar concerning interventions, patient populations, and pain intensity outcome measures, an indirect comparison method was deemed reasonable.

Limitations arising from our analysis approach should also be considered. For example, we defined and calculated a responder rate endpoint of 50% pain relief from the included datasets. While we selected this outcome to allow consistent comparison across studies, the Slangen and Petersen RCTs had study-defined primary endpoints that combined ≥ 50% pain relief with another measure. The Slangen study defined treatment success as meeting any 1 of 4 criteria (day or night pain relief ≥ 50% or Patient Global Impression of Change pain or sleep score ≥ 6). In the Petersen study, the criteria were narrower, with only a single pain threshold measure (≥50% pain relief) combined with no observed neurological deterioration. Our use of a different endpoint to the original study analyses may have introduced bias.

In addition, the necessity for data conversion in the LF-SCS studies may have impacted the fidelity of data pooling and between-study comparisons. Notably, Slangen et al., did not measure overall pain but instead assessed separate day and night pain in anticipation of a potential diurnal effect [39]; thus, we used the averaged pain intensity in our analysis, as per the Duarte et al., meta-analysis [55]. However, we cannot interpret whether individual patients in the Slangen study responded both during the day and night, nor how they would have rated their overall pain. As such, the averaged responder rate will likely represent the best-case LF-SCS treatment scenario for the Slangen RCT and its long-term follow-up observations.

The studies included in our review also had important shortcomings. Principally, none of the RCTs included a sham stimulation control arm. While this type of open-label study design is standard in the SCS field for practical reasons, it introduces a risk of bias and placebo effect. That said, the longer-term follow-up durations in the Petersen (12 months) and Slangen (60 months) RCTs should mitigate the placebo effect to a great extent. However, this mitigation may be weakened in the 60-month Slangen analysis due to the inclusion of an additional cohort and a high attrition rate (data were available from only 22 of 40 implanted patients, i.e., 55%) [42].

We also noted that the LF-SCS sample sizes in the Slangen and de Vos studies were small compared with the Petersen study; this may have resulted in the individual study comparisons between the modalities being underpowered. In addition, the de Vos study had nearly 4-fold the percentage of type 1 diabetes at baseline compared with the Petersen RCT. This large difference in type 1 diabetes prevalence may have contributed to the nonsignificant difference in responder rate between these 2 studies, the only comparison between 10 kHz SCS and LF-SCS that did not show a statistically significant difference.

Other considerations include a large number of centers in the 10 kHz SCS study compared with the LF-SCS trials and the time frame of study enrollment (Petersen et al., 2017–2019 [38]; Slangen et al., 2010–2013 [39]; de Vos et al., 2008–2012 [40]). In addition, the COVID pandemic may have impacted retainment and follow-up in the Petersen RCT.

### 4.4. Implications for Practice, Policy, and Future Research

In this systematic review, we compared results from 10 kHz SCS and LF-SCS for the treatment of lower limb pain due to PDN. While the RCT designs were largely consistent, the studies had different pain measurement approaches and minor inclusion/exclusion criteria variations. A future head-to-head study would provide a more consistent context for comparison and complement our analysis.

To the extent feasible, additional studies that develop the evidence base in the SCS field with potentially reduced risk of bias could also be helpful. Nonetheless, clinicians are most guided by rigorous data that provide evidence for selecting one treatment over another for a well-defined patient population. The results of our review indicate that patients with refractory PDN who receive 10 kHz SCS can have a significantly higher reduction in pain and a greater likelihood of response to therapy at 6 months than those who receive LF-SCS.

In any future study, as per the included RCTs, it would also be advantageous to incorporate other outcome measures that provide a more comprehensive evaluation of the patient’s experience of chronic pain—for example, HRQoL, function, medication use, and neurological and sensory changes. During our review, we noted that each study presented the 5-dimensional EuroQol (EQ-5D) overall health score (VAS) measured during the 6-month randomized phase. However, our analysis was focused on pain relief and did not include this outcome. Future systematic reviews should examine these results and the long-term HRQoL outcomes available from the Petersen [77] and Slangen [41] RCTs. In addition, future analyses should include a numeric synthesis of study-related AEs. To that end, it would be helpful if SCS studies reported AEs in a consistent way.

## 5. Conclusions

Spinal cord stimulation is increasingly used to address the debilitating symptoms of neuropathic pain in PDN patients. We identified RCT evidence supporting both 10 kHz SCS and LF-SCS as beneficial treatments for patients with PDN who fail conventional medical management. Our analysis indicates that 10 kHz SCS can provide superior pain relief and pain responder rate over LF-SCS for managing PDN patients. An RCT that randomizes PDN patients to either 10 kHz SCS or LF-SCS would be valuable for confirming these results.

## Figures and Tables

**Table 1:** Study characteristics.

	Study		
Characteristic	Petersen et al. [38]	Slangen et al. [39]	de Vos et al. [40]
Centers (Countries)	18 (USA)	2 (NL)	7 (NL, DK, BE, DE)
SCS modality	10 kHz SCS Paresthesia-free (Nevro)	LF-SCS Continuous paresthesia (Medtronic)	LF-SCS Continuous paresthesia (St. Jude Medical)
Comparison	10 kHz SCS + CMM vs. CMM	LF-SCS + CMM vs. CMM	LF-SCS + CMM vs. CMM
Blinding	No	No	No
Follow-up duration	6 mo	6 mo	6 mo
Indication, pain area	PDN, LL	PDN, LL	PDN, LE
Pain rating scale	VAS (0–10 cm)	NRS (0–10 points)	VAS (0–100 points)
Pain inclusion criteria	LL VAS ≥ 5 cm	LL NRS ≥ 5 points	LE VAS ≥ 50 points
Pain exclusion criteria	UL VAS ≥ 3 cm	UL NRS > 3 points	UE Pain > 20 points
Randomization ratio	1:1	3:2	2:1
Mean age by group	10 kHz SCS + CMM: 60.7 ± 11.4 y CMM: 60.8 ± 9.9 y	LF-SCS + CMM: 57.1 ± 12.4 y CMM: 56.5 ± 8.0 y	LF-SCS + CMM: 58 ± 11 y CMM: 61 ± 12 y
Female/male proportion by group	10 kHz SCS + CMM: 38%/62% CMM: 36%/64%	LF-SCS + CMM: 32%/68% CMM: 36%/64%	LF-SCS + CMM: 37.5%/62.5% CMM: 35%/65%
Diabetes Type I/II proportion by group	10 kHz SCS + CMM: 7%/93% CMM: 3%/97%	LF-SCS + CMM: 14%/86% CMM: 7%/93%	LF-SCS + CMM: 25%/75% CMM: 25%/75%
Diabetes durationby group	10 kHz SCS + CMM: 12.9 ± 8.5 y CMM: 12.2 ± 8.5 y	LF-SCS + CMM: 12.7 ± 10.1 y CMM: 12.6 ± 7.2 y	LF-SCS + CMM: 16 ± 11 y CMM: 17 ± 12 y
Duration of pain or peripheral neuropathyby group	10 kHz SCS + CMM: 7.4 ± 5.7 y ^‡^ CMM: 7.1 ± 5.1 y ^‡^	LF-SCS + CMM: 6.0 ± 5.1 y ^†^ CMM: 4.9 ± 3.6 y ^†^	LF-SCS + CMM: 7 ± 6 y ^†^ CMM: 7 ± 6 y ^†^
Duration of temporary stimulation trial	5–7 d	2 w	Up to 1 w
*n* randomized by group	10 kHz SCS + CMM: 113 CMM: 103	LF-SCS + CMM: 22 CMM: 14	LF-SCS + CMM: 40 CMM: 20
SCS group *n* trialed *n* with ≤50% pain relief *n* with ≥50% pain relief *n* implanted	104 6 98 90 ^#^	22 * 4 17 17	40 3 37 37
SCS group Proportion with a successful trial	94%	77%	92.5%
SCS group Trial-to-permanent implant proportion	87%	77%	92.5%

^†^ Duration of pain. ^‡^ Duration of peripheral neuropathy. * Trial lead could not be placed in 1 subject due to an adverse event. ^#^ 8 subjects had a successful trial but did not receive an implant due to declining the implant (*n* = 4), being lost-to-follow-up (*n* = 3), or having an adverse event (*n* = 1). Abbreviations: BE, Belgium; CMM, Conventional Medical Management; d, Day(s); DE, Germany; DK, Denmark; LE, Lower Extremities; LF-SCS, Traditional Low-Frequency Spinal Cord Stimulation; LL, Lower Limbs; mo, Month(s); NL, The Netherlands; NRS, Numerical Rating Scale; PDN, Painful Diabetic Neuropathy; SCS, Spinal Cord Stimulation; UE, Upper Extremities; UL, Upper Limbs; USA, United States of America; VAS, Visual Analog Scale; w, Weeks(s); y, Year(s).

**Table 2:** Comparison of the mITT population mean pain intensity reductions from baseline at 6 months.

		LF-SCS Group		
Statistic	10 kHz SCS [38]	Slangen LF-SCS ^‡^ [39]	de Vos LF-SCS [40]	Pooled LF-SCS ^†^ [39,40]
mITT population ^∫^	104	22	40	62
Mean baseline pain score	7.6	7.1	7.3	7.2
Mean reduction in pain score at 6 months (95% CI) *	−5.60 (−6.09, −5.11)	−2.73 (−4.02, −1.44)	−4.20 (−5.19, −3.21)	−3.42 (−3.95, −2.89)
*p*-value ^§^ 10 kHz SCS vs. LF-SCS group		*p* < 0.0001	*p* = 0.0060	*p* < 0.0001
Percentage reduction in pain relative to baseline	73.7%	38.7%	57.5%	47.5%

^∫^ Randomized individuals who entered the SCS trial phase; * LOCF was imputed for subjects lost to follow-up. ^§^ 10 kHz SCS vs. LF-SCS group, two-sample *t*-test. ^‡^ 6-month graphical format data extracted from Slangen et al. (2014) [39], Figure 2, using WebPlotDigitizer (https://automeris.io/WebPlotDigitizer, accessed 25 August 2022). ^†^ Source data: Slangen et al. (2014) [39] and de Vos et al. (2014) [40]. Abbreviations: CI, Confidence Interval; LF-SCS, Traditional Low-Frequency Spinal Cord Stimulation; LOCF, Last Observation Carried Forward; mITT, Modified Intention-to-Treat; SCS, Spinal Cord Stimulation.

**Table 3:** Comparison of the mITT population responder rates at 6 months.

		LF-SCS Group		
Statistic	10 kHz SCS [38]	Slangen LF-SCS [39]	de Vos LF-SCS [40]	Pooled LF-SCS [39,40]
Number of responders *	75	9	25	34
mITT population ^†^	104	22	40	62
Proportion of responders (%) ^‡^	72.12%	40.91%	62.50%	54.84%
RR (95% CI) 10 kHz SCS vs. LF-SCS group		1.76 (1.05, 2.95)	1.15 (0.88, 1.51)	1.32 (1.02, 1.70)
*p*-value ^§^		*p* = 0.016	*p* = 0.1478	*p* = 0.018

* Early withdrawal due to cause was considered a treatment failure; LOCF was imputed for subjects lost to follow-up. ^†^ Randomized individuals who entered the SCS trial phase; ^‡^ Proportion of the population with ≥50% reduction in pain intensity from baseline, based on a 0–10 pain scale. ^§^ Wald chi-square for RR = 1. Abbreviations: CI, Confidence Interval; mITT, Modified Intention-to-Treat; LF-SCS, Traditional Low-Frequency Spinal Cord Stimulation; LOCF, Last Observation Carried Forward; RR, Relative Risk; SCS, Spinal Cord Stimulation.

**Table 4:** Comparison of the permanent implant population responder rates at 6 months.

		LF-SCS Group		
Statistic	10 kHz SCS [38]	Slangen LF-SCS [39]	de Vos LF-SCS [40]	Pooled LF-SCS [39,40]
Number of responders *	75	9	25	34
Permanent implant Population ^†^	90	17	37	54
Proportion of responders (%) ^‡^	83.33%	52.94%	67.57%	62.96%
RR (95% CI) 10 kHz SCS vs. LF-SCS group		1.57 (1.00, 2.49)	1.23 (0.97, 1.57)	1.32 (1.06, 1.66)
*p*-value ^§^		*p* = 0.026	*p* = 0.044	*p* = 0.0072

* Early withdrawal due to cause was considered a treatment failure; LOCF was imputed for subjects lost to follow-up. ^†^ Subjects who completed the SCS trial phase and received a permanent system. ^‡^ Proportion of the population with ≥50% reduction in pain intensity from baseline, based on a 0–10 pain scale. ^§^ Wald chi-square for RR = 1. Abbreviations: CI, Confidence Interval; LF-SCS, Traditional Low-Frequency Spinal Cord Stimulation; LOCF, Last Observation Carried Forward; RR, Relative Risk; SCS, Spinal Cord Stimulation.

**Table 5:** Treatment-related adverse events.

	Study					
	Petersen et al. [38]	Petersen et al. ^†^ [50]	Slangen et al. [39]	van Beek et al. ^§^ [41]	van Beek et al. ^§‡^ [42]	de Vos et al. [40]
Follow-up Duration	6 mo	12 mo	6 mo	24 mo	60 mo	6 mo
*n*	90 implanted	154 implanted *	17 implanted	17 implanted	40 implanted	37 implanted
Postimplant infection *n* patients (%)	3 (3.3%)	8 (5.2%)	6 w: 1 (5.9%)	6 w: 1 (5.9%)	6 w and 2 mo: 2 (5.0%)	0 ⌃
Lead migration *n* patients (%)	1 (1.1%)	1 (0.6%)	-	-	-	1 (2.7%)
IPG site pain *n* patients	1 (1.1%)	-	-	-	10 (25.0%)	2 (5.4%)
Uncomfortable stimulation *n* patients (%)	1 (1.1%)	-	-	-	9 (22.5%)	-
Lead revision *n* patients (%)	-	1 (0.6%)	-	4 (23.5%)	4 (10.0%)	Due to lead migration: 1 (2.7%) Due to incomplete paresthesia overlap: 2 (5.4%)
Lead replacement *n* patients (%)	-	-	-	-	3 (7.5%)	-
IPG revision *n* patients (%)		2 (1.3%)	-	»	Due to prolonged pocket pain (20 mo): 1 (2.5%)	Due to IPG site pain: 2 (5.4%)
IPG explant due to AE *n* patients (%)	2 (2.2%)	Due to infection: 5 (3.2%)	Due to postimplant infection: 1 (5.9%)	Due to postimplant infection: 1 (5.9%)	Due to infection: 2 (5.0%) Due to lack of efficacy: 6 (15.0%)	-
IPG replacement *n* patients (%)	0 ^ψ^	0 ^ψ^	0 ^ψ^	1 × replacement: 2 (11.8%)	1 × replacement: 8 (20.0%) 2 × replacements: 5 (12.5%)	
Other *n* patients (%)	Wound dehiscence: 2 (2.2%) Impaired healing: 1 (1.1%) Device extrusion: 1 (1.1%) Incision site pain: 1 (1.1%) Other: 7 (7.8%) ^∫^	-	Dural puncture with subdural hematoma sequala, leading to death: 1 (5.9%)	-	-	Coagulopathy during implantation: 1 (2.7%)

^†^ Additional follow-up to Petersen et al. (2021) [38]. * Includes 90 initial implants and 64 crossover implants. ^§^ Additional follow-up to Slangen et al. (2014) [39]. ^‡^ Includes crossovers and *n* = 12 from Pluijms et al. (2012) [31]. ^Ψ^ No replacements reported. ^∫^ *n* = 1 each for contact dermatitis, urticaria, radiculopathy, gastroesophageal reflux, myalgia, arthralgia, and hyporeflexia. ⌃ *n* = 1 infection during the trial phase. Abbreviations: AE, Adverse Event; IPG, Implantable Pulse Generator; mo, Month(s); w, Weeks(s).

## Data Availability

Not applicable.

## Supplementary Materials

The following supporting information can be downloaded at: https://www.mdpi.com/article/10.3390/biomedicines10102630/s1, Supplementary Material: Table S1. PubMed search strategy; Table S2. CENTRAL search strategy; Table S3. Cochrane Risk of Bias Assessment (Performed Using the RoB 2.0 Tool).
