# Supplementary material for: Indirect Comparison of 10 kHz Spinal Cord Stimulation (SCS) versus Traditional Low-Frequency SCS for the Treatment of Painful Diabetic Neuropathy: A Systematic Review of Randomized Controlled Trials
Source: Biomedicines. 2022 Oct 19;10(10):2630. doi: 10.3390/biomedicines10102630 (PMC9599433; doi:10.3390/biomedicines10102630)
Supplement: Supplementary file 1 [file biomedicines-10-02630-s001.zip › Supplementary Material 1_26Aug2022.pdf]

Supplementary Material 1

Table S1. PubMed search strategy.

Search ID	Search term
#1	randomized controlled trial[pt]
#2	controlled clinical trial[pt]
#3	randomized[tiab]
#4	placebo[tiab]
#5	drug therapy[sh]
#6	randomly[tiab]
#7	trial[tiab]
#8	groups[tiab]
#9	#1 OR #2 OR #3 OR #4 OR #5 OR #6 OR #7 OR #8
#10	animals[mh] NOT humans[mh]
#11	#9 NOT #10
#12	clinical trials, phase iii[MeSH Terms]
#13	"Phase 3"[tiab] or "phase3"[tiab] or "phase III"[tiab] or P3[tiab] or "PIII"[tiab]
#14	#12 OR #13
#15	#11 OR #14
#16	"spinal cord"[tiab] OR spine[tiab] OR spinal[tiab] OR epidural[tiab] OR "dorsal column*"[tiab]
#17	stimulation[tiab] OR stimulator[tiab] OR neuromodulation[tiab] OR neurostimulator[tiab]
#18	#16 AND #17
#19	"spinal cord stimulation"[mesh] OR "electric stimulation therapy"[mesh] OR scs[tiab]
#20	#18 OR #19
#21	"Diabetic Neuropathies"[mesh]
#22	diabet*[tiab] AND (neuropath*[tiab] OR polyneuropath*[tiab])
#23	#21 OR #22
#24	#20 AND #23
#25	#15 AND #24

Table S2. CENTRAL search strategy.

Search ID	Search term
#1	"spinal cord":ti,ab OR spine:ti,ab OR spinal:ti,ab OR epidural:ti,ab OR "dorsal column*":ti,ab
#2	stimulation:ti,ab OR stimulator:ti,ab OR neuromodulation:ti,ab OR neurostimulator:ti,ab
#3	#1 AND #2
#4	MeSH descriptor: [Spinal Cord Stimulation] explode all trees
#5	MeSH descriptor: [Electric Stimulation Therapy] explode all trees
#6	scs:ti,ab
#7	#4 OR #5 OR #6
#8	#3 OR #7
#9	MeSH descriptor: [Diabetic Neuropathies] explode all trees
#10	diabet*:ti,ab AND (neuropath*:ti,ab OR polyneuropath*:ti,ab)
#11	#9 OR #10
#12	#8 AND #11 in Trials
