# Supplementary material for: Indirect Comparison of 10 kHz Spinal Cord Stimulation (SCS) versus Traditional Low-Frequency SCS for the Treatment of Painful Diabetic Neuropathy: A Systematic Review of Randomized Controlled Trials
Source: Biomedicines. 2022 Oct 19;10(10):2630. doi: 10.3390/biomedicines10102630 (PMC9599433; doi:10.3390/biomedicines10102630)
Supplement: Supplementary file 1 [file biomedicines-10-02630-s001.zip › Supplementary Material 2_26Aug2022.pdf]

Supplementary Material 2

Table S3. Cochrane Risk of Bias Assessment (Performed Using the RoB 2.0 Tool)

**Unique ID**	Petersen et al (2021)	**Study ID**	Petersen et al (2021)	**Assessor**	DE
**Ref or Label**	Petersen et al (2021)	**Aim**	assignment to intervention (the 'intention-to-treat' effect)		
**Experimental**	Conventional medical management (CMM) with adjunctive high-frequency 10 kHz SCS therapy	**Comparator**	Conventional medical management (CMM) alone	**Source**	Journal article(s)
**Outcome**	6 months: Pain intensity; Responder rate (at least 50% pain reduction)	**Results**		**Weight**	1

Domain	Signalling question	Response	Comments
**Bias arising from the randomization process**	1.1 Was the allocation sequence random?	Y	Computer generated randomization.
	1.2 Was the allocation sequence concealed until participants were enrolled and assigned to interventions?	Y	Randomization was concealed.
	1.3 Did baseline differences between intervention groups suggest a problem with the randomization process?	N	Baseline characteristics appear to be well-balanced
	**Risk of bias judgement**	**Low**	
**Bias due to deviations from intended interventions**	2.1.Were participants aware of their assigned intervention during the trial?	Y	Open-label study without blinding of participants or study personnel, or sham control
	2.2.Were carers and people delivering the interventions aware of participants' assigned intervention during the trial?	Y	
	2.3. If Y/PY/NI to 2.1 or 2.2: Were there deviations from the intended intervention that arose because of the experimental context?	PN	No deviations were reported.
	2.4 If Y/PY to 2.3: Were these deviations likely to have affected the outcome?	NA	
	2.5. If Y/PY/NI to 2.4: Were these deviations from intended intervention balanced between groups?	NA	
	2.6 Was an appropriate analysis used to estimate the effect of assignment to intervention?	Y	An ITT approach is reported, although there were 8 exclusions after exposure to the treatment during the screening trial.
	2.7 If N/PN/NI to 2.6: Was there potential for a substantial impact (on the result) of the failure to analyse participants in the group to which they were randomized?	NA	
	**Risk of bias judgement**	**Low**	
**Bias due to missing outcome data**	3.1 Were data for this outcome available for all, or nearly all, participants randomized?	N	Low level of missing data at 6 months: 10 kHz SCS plus CMM: 15%, 17 of 113; CMM: 9%, 9 of 103.
	3.2 If N/PN/NI to 3.1: Is there evidence that result was not biased by missing outcome data?	N	The level of missing data was not balanced between the arms. In addition, the missing participants were excluded from the 6-month analysis.
	3.3 If N/PN to 3.2: Could missingness in the outcome depend on its true value?	PN	Considering the 10 kHz SCS group, there were 8 exclusions after exposure to the treatment during the screening trial. The published article does not provide sufficient detail to confirm that the exclusions were not related to the pain outcome. However, individual patient data (IPD) confirmed that all 8 patients completed the trial, all were responders, and 6 had at least 75% pain relief; this suggests that the exclusions were probably not related to the pain outcome.
	3.4 If Y/PY/NI to 3.3: Is it likely that missingness in the outcome depended on its true value?	NA	
	**Risk of bias judgement**	**Low**	
**Bias in measurement of the outcome**	4.1 Was the method of measuring the outcome inappropriate?	N	VAS is a validated pain intensity scale
	4.2 Could measurement or ascertainment of the outcome have differed between intervention groups?	N	
	4.3 Were outcome assessors aware of the intervention received by study participants?	Y	VAS score was patient-reported outcome (subjective scale). Since the trial was open-label in design, the assessor (ie, the patient) was aware of the treatment assignment.
	4.4 If Y/PY/NI to 4.3: Could assessment of the outcome have been influenced by knowledge of intervention received?	Y	Since the patient was aware of the intervention, they may have been influenced by expecting a benefit from 10 kHz SCS, or not perceiving CMM as a proper treatment. In addition, the patient was aware of being allowed to crossover at 6 months.
	4.5 If Y/PY/NI to 4.4: Is it likely that assessment of the outcome was influenced by knowledge of intervention received?	PY	
	**Risk of bias judgement**	**High**	
**Bias in selection of the reported result**	5.1 Were the data that produced this result analysed in accordance with a pre-specified analysis plan that was finalized before unblinded outcome data were available for analysis?	PY	A summary of the trial protocol was published by Mekhail et al (2020). A statistical analysis plan (SAP) is also publicly available. However, it is not confirmed that the final SAP preceded the availability of unblinded outcome data to the trial investigators.
	5.2 ... multiple eligible outcome measurements (e.g. scales, definitions, time points) within the outcome domain?	PN	No apparent indications of reporting selectivity at 6 months from examination of the protocol and SAP.
	5.3 ... multiple eligible analyses of the data?	PN	
	**Risk of bias judgement**	**Low**	
**Overall bias**	**Risk of bias judgement**	**High**	

Unique ID	Slangen et al (2014)	Study ID	Slangen et al (2014)	Assessor	DE
Ref or Label	Slangen et al (2014)	Aim	assignment to intervention (the 'intention-to-treat' effect)		
Experimental	Best medical therapy with adjunctive LF-SCS therapy	Comparator	Best medical therapy (BMT) alone	Source	Journal article(s); Non-commercial trial registry record (e.g. ClinicalTrials.gov record)
Outcome	6 months: Pain intensity; Responder rate (at least 50% pain reduction)	Results		Weight	1

Domain	Signalling question	Response	Comments
Bias arising from the randomization process	1.1 Was the allocation sequence random?	Y	Computer generated randomization.
	1.2 Was the allocation sequence concealed until participants were enrolled and assigned to interventions?	NI	No information is provided regarding concealment; however, it is likely that the allocation was concealed since computer randomization was used.
	1.3 Did baseline differences between intervention groups suggest a problem with the randomization process?	N	Baseline characteristics appear to be well-balanced.
	**Risk of bias judgement**	**Low**	
Bias due to deviations from intended interventions	2.1.Were participants aware of their assigned intervention during the trial?	Y	Open-label study without blinding of participants or study personnel.
	2.2.Were carers and people delivering the interventions aware of participants' assigned intervention during the trial?	Y	
	2.3. If Y/PY/NI to 2.1 or 2.2: Were there deviations from the intended intervention that arose because of the experimental context?	PN	No deviations were reported.
	2.4 If Y/PY to 2.3: Were these deviations likely to have affected the outcome?	NA	
	2.5. If Y/PY/NI to 2.4: Were these deviations from intended intervention balanced between groups?	NA	
	2.6 Was an appropriate analysis used to estimate the effect of assignment to intervention?	Y	ITT analysis was performed
	2.7 If N/PN/NI to 2.6: Was there potential for a substantial impact (on the result) of the failure to analyse participants in the group to which they were randomized?	NA	
	**Risk of bias judgement**	**Low**	
Bias due to missing outcome data	3.1 Were data for this outcome available for all, or nearly all, participants randomized?	Y	Low level of missing data from each arm, but not balanced between the arms: LF-SCS 14% (3 of 22); CMM 7% (1 of 14). However, missing participants were treated as nonresponders.
	3.2 If N/PN/NI to 3.1: Is there evidence that result was not biased by missing outcome data?	NA	
	3.3 If N/PN to 3.2: Could missingness in the outcome depend on its true value?	NA	
	3.4 If Y/PY/NI to 3.3: Is it likely that missingness in the outcome depended on its true value?	NA	
	**Risk of bias judgement**	**Low**	
Bias in measurement of the outcome	4.1 Was the method of measuring the outcome inappropriate?	N	NRS is a validated pain intensity scale
	4.2 Could measurement or ascertainment of the outcome have differed between intervention groups?	N	
	4.3 Were outcome assessors aware of the intervention received by study participants?	Y	NRS score was patient-reported outcome (subjective scale). Since the trial was open-label in design, the assessor (ie, the patient) was aware of the treatment assignment.
	4.4 If Y/PY/NI to 4.3: Could assessment of the outcome have been influenced by knowledge of intervention received?	Y	Since the patient was aware of the intervention, they may have been influenced by expecting a benefit from LF-SCS, or not perceiving BMT as a proper treatment.
	4.5 If Y/PY/NI to 4.4: Is it likely that assessment of the outcome was influenced by knowledge of intervention received?	PY	
	**Risk of bias judgement**	**High**	
Bias in selection of the reported result	5.1 Were the data that produced this result analysed in accordance with a pre-specified analysis plan that was finalized before unblinded outcome data were available for analysis?	NI	Analysis intentions are not available, ie, no published protocol or statistical analysis plan
	5.2 ... multiple eligible outcome measurements (e.g. scales, definitions, time points) within the outcome domain?	PN	No apparent indications of reporting selectivity. The use of dichotomous responder status is standard in the SCS field (ie, not selective reporting). The analysis used the standard definition of treatment response (at least 50% pain reduction).
	5.3 ... multiple eligible analyses of the data?	PN	
	**Risk of bias judgement**	**Some concerns**	
Overall bias	**Risk of bias judgement**	**High**	

Unique ID	de Vos et al (2014)	Study ID	de Vos et al (2014)	Assessor	DE
Ref or Label	de Vos et al (2014)	Aim	assignment to intervention (the 'intention-to-treat' effect)		
Experimental	Best medical therapy with adjunctive LF-SCS therapy	Comparator	Best medical therapy (BMT) alone	Source	Journal article(s); Non-commercial trial registry record (e.g. ClinicalTrials.gov record)
Outcome	6 months: Pain intensity; Responder rate (at least 50% pain reduction)	Results		Weight	1

Domain	Signalling question	Response	Comments
Bias arising from the randomization process	1.1 Was the allocation sequence random?	Y	Block stratified randomization is likely to be computer based.
	1.2 Was the allocation sequence concealed until participants were enrolled and assigned to interventions?	NI	No information is provided regarding concealment; however, it is likely that the allocation was concealed if computer randomization was used.
	1.3 Did baseline differences between intervention groups suggest a problem with the randomization process?	N	Baseline characteristics appear to be well-balanced (no statistically significant differences between the groups).
	**Risk of bias judgement**	**Low**	
Bias due to deviations from intended interventions	2.1.Were participants aware of their assigned intervention during the trial?	Y	Open-label study without blinding of participants or study personnel.
	2.2.Were carers and people delivering the interventions aware of participants' assigned intervention during the trial?	Y	
	2.3. If Y/PY/NI to 2.1 or 2.2: Were there deviations from the intended intervention that arose because of the experimental context?	PN	No deviations were reported.
	2.4 If Y/PY to 2.3: Were these deviations likely to have affected the outcome?	NA	
	2.5. If Y/PY/NI to 2.4: Were these deviations from intended intervention balanced between groups?	NA	
	2.6 Was an appropriate analysis used to estimate the effect of assignment to intervention?	Y	ITT analysis was performed
	2.7 If N/PN/NI to 2.6: Was there potential for a substantial impact (on the result) of the failure to analyse participants in the group to which they were randomized?	NA	
	**Risk of bias judgement**	**Low**	
Bias due to missing outcome data	3.1 Were data for this outcome available for all, or nearly all, participants randomized?	Y	Low and balanced level of missing data in each arm: LF-SCS 10%, 4 of 40; BMT 10%, 2 of 20. The authors presented an ITT analysis.
	3.2 If N/PN/NI to 3.1: Is there evidence that result was not biased by missing outcome data?	NA	
	3.3 If N/PN to 3.2: Could missingness in the outcome depend on its true value?	NA	
	3.4 If Y/PY/NI to 3.3: Is it likely that missingness in the outcome depended on its true value?	NA	
	**Risk of bias judgement**	**Low**	
Bias in measurement of the outcome	4.1 Was the method of measuring the outcome inappropriate?	N	VAS is a validated pain intensity scale
	4.2 Could measurement or ascertainment of the outcome have differed between intervention groups?	N	
	4.3 Were outcome assessors aware of the intervention received by study participants?	Y	VAS score was patient-reported outcome (subjective scale). Since the trial was open-label in design, the assessor (ie, the patient) was aware of the treatment assignment.
	4.4 If Y/PY/NI to 4.3: Could assessment of the outcome have been influenced by knowledge of intervention received?	Y	Since the patient was aware of the intervention, they may have been influenced by expecting a benefit from LF-SCS, or not perceiving BMT as a proper treatment. In addition, the patient was aware of being allowed to crossover at 6 months.
	4.5 If Y/PY/NI to 4.4: Is it likely that assessment of the outcome was influenced by knowledge of intervention received?	PY	
	**Risk of bias judgement**	**High**	
Bias in selection of the reported result	5.1 Were the data that produced this result analysed in accordance with a pre-specified analysis plan that was finalized before unblinded outcome data were available for analysis?	NI	Analysis intentions are not available, ie, no published protocol or statistical analysis plan
	5.2 ... multiple eligible outcome measurements (e.g. scales, definitions, time points) within the outcome domain?	PN	No apparent indications of reporting selectivity. The use of dichotomous responder status is standard in the SCS field (ie, not selective reporting). The analysis used the standard definition of treatment response (at least 50% pain reduction).
	5.3 ... multiple eligible analyses of the data?	PN	
	**Risk of bias judgement**	**Some concerns**	
Overall bias	**Risk of bias judgement**	**High**	
